# Mental Health Service Users and Their Caregivers Perspectives on Personal Recovery from Severe Mental Health Conditions in Cape Town, South Africa: A Qualitative Study

**DOI:** 10.1007/s40737-023-00341-8

**Published:** 2023-04-15

**Authors:** Fadia Gamieldien, Roshan Galvaan, Bronwyn Myers, Katherine Sorsdahl

**Affiliations:** 1 Department of Psychiatry and Mental Health, Alan J. Flisher Centre for Public Mental Health, University of Cape Town, 46 Sawkins Road, Rondebosch, Cape Town, 7700 South Africa; 2 Division of Occupational Therapy, Department of Health and Rehabilitation Sciences, University of Cape Town, Cape Town, South Africa; 3 Inclusive Practices Africa Research Unit, Department of Health and Rehabilitation Sciences, University of Cape Town, Cape Town, South Africa; 4 Curtin enAble Institute, Faculty of Health Sciences, Curtin University, Perth, WA Australia; 5 Alcohol, Tobacco and Other Drug Research Unit, South African Medical Research Council, Cape Town, South Africa; 6 Division of Addiction Psychiatry, Department of Psychiatry and Mental Health, University of Cape Town, Cape Town, South Africa

**Keywords:** Recovery, Severe mental health conditions, Low-and-middle-income countries, Mental health service users, Caregivers

## Abstract

Severe mental health conditions (SMHCs) significantly contribute to the global disease burden. In low-and-middle-income countries (LMICs) like South Africa, the long-term impact of SMHCs on individuals and their families is serious. However, mental health services focus on clinical recovery, with little attention given to the personal recovery needs of mental health service users (MHSUs) and their caregivers. The CHIME framework outlines five domains characterising personal recovery: connectedness, hope and optimism about the future, identity, meaning in life, and empowerment. This qualitative, descriptive study sought insights from male MHSUs and their caregivers on their perspectives of personal recovery from SMHCs. Four male MHSUs and three of their caregivers were purposively selected from Cape Flats communities in the Western Cape. Data were collected using visual participatory methods, including photovoice, life graphs, community maps, and photo-elicitation interviews with MHSUs. In addition, semi-structured interviews were held with caregivers. Data were thematically analysed, and two main themes emerged: Finding meaningful participation and affirming agency. These themes describe how diverse contextual, socioeconomic, political, demographic, cultural, and spiritual factors help and hinder personal recovery. MHSUs and their caregivers sought support from mental health non-profit organisations (MH-NPOs) because of stigmatising attitudes from their communities. MH-NPOs provided MHSUs with long-term relational support and opportunities to build their capacities which helped them access living, learning, working and socialising opportunities. Understanding the diverse needs of MHSUs and including MH-NPOs in scaling up community-based mental health services in LMICs will enable more accessible services that support personal recovery.

## Introduction

Severe mental health conditions (SMHCs), including schizophrenia spectrum disorders, psychotic disorders, and bipolar affective disorders, contribute significantly to the global disease burden (Charlson et al., 2018; Rehm & Shield, 2019). In low- and-middle-income countries (LMICs) such as South Africa (SA), many people with SMHCs are undetected and do not receive the treatment they need (Demyttenaere et al., 2004; Kohn et al., 2004). This treatment gap is further exacerbated because people living with SMHCs require complex interventions to address their social and economic difficulties (Charlson et al., 2018). Additionally, the long-term impact on individuals and their families is substantial (Docrat et al., 2019; Vigo et al., 2016). In the public sector, mental health care for SMHCs is rendered predominantly through in-patient specialist psychiatric services (Docrat et al., 2019) with limited availability of community services. In addition, available services tend to focus on clinical recovery, giving little attention to the personal recovery needs of mental health service users (MHSUs) (Gamieldien et al., 2022; Kleintjes et al., 2013).

Both clinical and personal recovery are critical components of mental health service provision. However, clinical recovery tends to be limited as it focuses on treating SMHCs symptoms, with the clinician positioned as the expert (Gamieldien et al., 2022; Llewellyn-Beardsley et al., 2019; Rennick-Egglestone et al., 2019; Slade, 2009). While personal recovery values clinical recovery (Slade, 2009), it focuses on experiences and actions taken to live a meaningful life with SMHCs (Anthony, 1993; Chamberlin, 2007; Deegan, 1988). Furthermore, personal recovery positions MHSUs as experts by experience who should inform the design and implementation of mental health services (Kakuma et al., 2010; Ørjasæter & Almvik, 2022; Sullivan et al., 2017). To better describe the complexity of the recovery process several frameworks have been developed. One such framework is the widely endorsed CHIME Framework (Leamy et al., 2011; Leendertse et al., 2021).

The CHIME Framework was conceptualised by Leamy et al. (2011) and consists of five domains used to organise the dimensions of recovery. The CHIME framework consists of a network of interlinking concepts to operationalise recovery (Leamy et al., 2011; Leendertse et al., 2021; Vogel et al., 2020). These domains are connectedness, hope and optimism about the future, identity, meaning in life and empowerment. CHIME was developed following a systematic review and narrative synthesis on conceptualisations of personal recovery from mental illness, which included 97 papers from 13 high-income countries (HICs). Leamy et al. (2011) acknowledge the limitations of CHIME and its applicability in diverse settings. They advocate for more research with culturally diverse ethnic groups because the black and minority ethnic (BME) groups included in their study had different perspectives on recovery. The emphasis placed on family and community involvement, spirituality, and overcoming stigma highlights that culture-specific factors in BME groups differ from the views on recovery from non-BME (Brijnath, 2015; Leamy et al., 2011; van Weeghel et al., 2019) or HIC perspectives. There is a need to better understand how contextual barriers and access to resource influences recovery in LMICs.

Research on personal recovery has mainly emerged from the perspectives of HICs, with few descriptions emerging on personal recovery from LMIC perspectives (Gamieldien et al., 2021a). A recent scoping review (Gamieldien et al., 2021a) emphasised the relational aspect of personal recovery for MHSU from LMICs. This was less salient in reviews of personal recovery for MHSUs in HICs (Mathew et al., 2018). Although numerous studies advocate for a greater understanding of how personal recovery occurs for MHSUs in LMICs, including in South Africa (De Wet & Pretorius, 2020; Docrat et al., 2019), studies on the lived experience and contextual factors impacting MHSUs personal recovery journey remain sparse (Gamieldien et al., 2022). There has been little exploration of MHSUs exeriences, or the systemic and socio-economic barriers MHSUs face to personal recovery in their communities (Gamieldien, Galvaan, & Duncan, 2021a; Gamieldien et al., 2022). This is important to build personal recovery assets and services which address the diverse needs of MHSUs.

Given that SMHCs are characterised by gender differences and behavioural problems that vary between men and women (Vázquez-Reyes et al., 2021), adopting a gendered perspective to explore personal recovery may help tailor personal recovery supports for the different needs of men and women (Smith et al., 2018; Vázquez-Reyes et al., 2021). Gender is important to consider since men are less likely to seek help for mental health difficulties, resulting in an under-utilisation of services (Sagar-Ouriaghli et al., 2019). Although specialist psychiatric hospitals in SA allocate more emergency in-patient beds for men, how men with SMHCs navigate their recovery once they return to their communities has not been explored (Botha et al., 2020). This impacts our ability to design interventions to support the personal recovery journeys of men with SMHCs. This study explored and described the views on personal recovery and access to services from the perspectives of men with SMHCs and their caregivers in Cape Town, South Africa.

### Study Context

This study was situated in Cape Town, a densely populated city in South Africa. Cape Town is a diverse city which includes the poorly resourced Cape Flats, a vast region outside the city centre notorious for a high prevalence of poverty, gangsterism, and violence (Bowers Du Toit, 2014; Mncube & Madikizela-Madiya, 2014). During apartheid, people designated as Black and Coloured^1^ were forcibly moved to the Cape Flats. Due to the demand for psychiatric beds, MHSUs residing on the Cape Flats have poor access to the three psychiatric hospitals that provide specialist, in-patient services for SMHCs in the Western Cape (Docrat et al., 2019). After being discharged from the hospital, MHSUs receive follow-up clinical care through psychiatric hospital outpatient departments or local community health centres (CHCs). MHSUs receive their psychotropic medication at these sites and are monitored for medication adherence. They do not receive any other services at this level of care as efforts to integrate mental health into primary healthcare remains slow (Jacob & Coetzee, 2018). To address this treatment gap, a few mental health non-profit organisations (MH-NPOs) offer community based mental health services. Staff at these MH-NPOs include health professionals, auxiliary workers, community members and MHSUs. MH-NPOs provide supported living, supported employment, daily activity-based programmes, support groups and in-hospital activities not included in the hospital rehabilitation programme (Grobbelaar, 2010). These services are inaccessible to the majority of MHSUs since they are mostly situated in areas that are far from the communities where MHSUs live. Those MHSUs who access the services benefit from the long term relational support that they need once discharged from hospital (Gamieldien et al., 2022).

### Research Design

This study uses a qualitative, descriptive research design (Bradshaw et al., 2017; Lambert & Lambert, 2012; Neergaard et al., 2009) to explore recovery from SMHCs from the vantage point of male MHSUs from Cape Flats communities. Photovoice (Wang & Burris, 1997) was implemented as a visual participatory method to provide insight into user experiences. Visual participatory methods are recognised in Public health research as helpful for co-producing knowledge in under-researched areas and with marginalised groups of people (Anderson Clarke & Warner, 2016; Cabassa et al., 2013; Lal et al., 2012; Wang & Redwood-Jones, 2001). The study reported on in this paper uses the Standards for Reporting Qualitative Research (SRQR) framework to enhance the transparency of the research (O’Brien et al., 2014).

### Researcher Characteristics

The first author, FG, is a woman of colour who lives on the Cape Flats. Her living experiences on the Cape Flats provided her with an intuitive understanding of the disadvantaged socio-economic communities in which participants lived. FG’s work experience first as a public sector, mental health occupational therapist exposed her to a gendered perspective on recovery from SMHCs. Second, in her prior qualitative research experience with male MHSUs, she identified that residential rehabilitation services in the Western Cape inadequately prepared MHSUs for community integration (Gamieldien, Galvaan, & Duncan, 2021a). Adding to these work experiences, her personal and professional commitment to human rights contributed to her strong belief in advocacy and self-representation of MHSUs. FG was especially interested in learning more about, and giving voice to MHSUs perspectives on recovery. FG’s extensive work experience in the sector positioned her well for this study as she was very familiar with MH-NPOs servicing the Western Cape. She also knew some of the Programme Managers offering community-based mental health services as a result of working together previously or, being involved in similar networks.

### Sample Selection and Recruitment

The following inclusion criteria were applied to purposively select eligible participants: (a) males aged between 18–60 years, (b) able to communicate in English or Afrikaans^2^ (c) diagnosed with an SMHC, (d) registered on the public health services database, and (e) living in a Cape Flats community. Maximum variation sampling techniques (Creswell & Poth, 2018) were used to ensure the sample included diverse perspectives and a range of recovery experiences. The factors considered in maximum variation sampling were age, race, residential circumstances, employment status and time since relapse. MHSUs were invited to identify caregiver participants. Caregivers had to be connected to the MHSUs and MHSUs had to consent to the caregivers participation. The inclusion criteria was thus that the MHSU identified the caregiver as fulfilling this role in their lives.

All participants were recruited via an MH-NPO. The Programme Manager at the MH-NPO is a social worker by profession and is bound to ethical and professional conduct. She identified MHSUs who met the inclusion criteria and explained the study to them. If they expressed an interest, she gained their consent to share their contact information with FG. Once FG had their details she telephoned them to explain the study further and gain consent for their participation in the study.

Of the 12 MHSUs who were eligible, four MHSUs consented to participate. Two MHSUs were not contactable at the time of recruitment. Six MHSUs declined to participate because of personal reasons they did not elaborate upon (n = 4), and caregivers disapproved of their involvement (n = 2). MHSUs were invited to nominate their caregivers to participate in the study. Three caregivers, associated with two of the MHSU participants, agreed to participate. Two MHSUs did not have caregivers whom they felt comfortable nominating. A total of seven participants (four MHSUs and three caregivers) were recruited for this study.

Key characteristics of the four MHSUs and their caregivers are presented in Table 1.

**Table 1:** Description of participants (MHSUs)

Participant ‘s Pseudonym	Age	Age at first psychiatric hospital admission	Year of the last psychiatric hospital admission (Range 9–20 years)	Living situation	Race	Employment status	Relationship status	History of Substance use	Caregivers
Patrick	50	21	2005	In supported housing with other MHSUs since 2006	Coloured	Employed as a cook in the education sector	Single, never married	Yes	Parents (Patrick’s Mother and Patrick’s Father)
Globie^a^	38	22	2012	In a family home on the Cape Flats with parents and siblings	Coloured	Employed as a shop assistant in the retail sector	Single, never married	Yes	Globie’s Mother
Siya	42	23	2008	In supported Housing with other MHSUs since 2012	Black	Unemployed	Single, never married	Yes	None identified
Reggie	50	23	2000	In supported Housing with other MHSUs since 2000	Coloured	Employed as a supervisor in a supported employment project for adults with intellectual disabilities in the NPO sector	Single, divorced	No	None identified

^a^Globe is a synonym for a lightbulb. “Globie” is a colloquial Afrikaans word for a globe. Globie is the nickname of this participant

Pseudonyms are used to protect participants' identities

The time elapsed since participants last hospital admissions ranged between nine-20 years

At the time of the study, all participants were single. Only one participant had ever been married

## Procedure

Data collection was conducted by FG, who has experience conducting qualitative research. The visual methods (Glaw et al., 2017) used included focused life graphs (Adriansen, 2012; Denicoff et al., 2002), production of a community map and photovoice methods (Wang & Burris, 1997). A life graph was used to visually represent significant events related to living with SMHCs (Adriansen, 2012; Denicoff et al., 2002). A community map is a drawing that shows places available in a sociocultural context (Sweet et al., 2018). Photo-voice methods involved MHSUs taking photos (Anderson Clarke & Warner, 2016; Cabassa et al., 2013; Wang & Burris, 1997) in their communities that express their personal recovery stories. These stories were discussed as part of a photo-elicitation interview. These methods were piloted with an MHSU (MM) who was part of the study’s advisory committee.

First, FG conducted an in-depth interview with each MHSU, during which the MHSU produced a life graph. By referring to the life-graph, participants were able to share important events in their lives such as (a) onset of mental illness; (b) experience of living with a mental illness; (c) significant life events; (d) exploration of substance use; (e) their access to psychiatric services, and (f) their future hopes and plans.

In the follow-up interview, participants discussed their recovery using their life graph as a prompt. After that, they were asked to develop a community map in preparation for the photovoice exercise. Participants were reluctant to draw their maps in the session because they wanted more time on their own to think about crafting their maps. This led them to use the time during the session to gather their ideas and write down places of interest and to use this as prompts when they took their photos. The community map assisted participants with identifying the places, people, and activities that contributed to their recovery experiences. They could use this map to plan what photos to take to depict their recovery.

MHSUs captured their recovery stories using a digital camera (Wang & Burris, 1997). Before issuing the digital cameras to participants, the researcher discussed possible ethical issues that could emerge during the photovoice process, including the need to obtain consent from people being photographed. MHSUs were shown how to use the digital camera. MHSUs had 5 weeks to take their pictures, allowing sufficient time to capture personally meaningful and contextually rich images of their recovery stories (Han & Oliffe, 2016). They brought these pictures to subsequent photo-elicitation interviews where each participant explained their stories (Piat et al., 2017). MHSUs were issued digital copies of their photographs and colour prints of their requested photos. The digital cameras were returned to FG.

FG also conducted semi-structured interviews in the homes of consenting caregivers. Using an interview guide, FG elicited discussion centred around living with someone with a mental illness, onset and duration of illness, access to psychiatric services for the MHSU and MHSU and caregiver access to support in and outside of their communities. They were also asked about MHSUs substance use histories and their (caregivers) understanding of recovery. They were probed about the factors that they think help or hinder recovery in MHSUs. All interviews lasted approximately 1 h and were audio-recorded and transcribed verbatim. Interviews conducted in Afrikaans were translated into English after transcription. Ethical approval was obtained from the Health Sciences Research Ethics Committee (HREC 655/2018) at a large university in the Western Cape and the Western Cape Province's Health Research Committee (WC_201902_010).

### Analysis

NVivo12 software was used to manage the data. Thematic data analysis (Creswell & Poth, 2018) guided engagement with the data. FG read the transcripts and reviewed the visual data sources to identify initial codes and preliminary themes. The second author (RG) and (FG) met regularly to refine the codes, categories, sub-themes and themes (Creswell & Poth, 2018; Saldaña, 2016) and reflexively analyse and interpret the data (Braun & Clarke, 2019). Themes were checked with MHSUs, as part of member checking. FG met with participants individually after the data was analysed so that they could give feedback on the themes which emerged. This increased confidence in our interpretation of the data (Nowell et al., 2017). FG kept a research journal to promote reflexivity to document her thoughts and insights on personal recovery during the interview and coding process.

## Results

Two main themes were identified: "Finding meaningful participation” and “Affirming agency”. These two themes with corresponding sub-themes are represented in Table 2 and discussed in detail in this section.

**Table 2:** Overview of findings

Theme 1	Sub-theme
Finding meaningful participation	1.1 Developing self-management strategies
	1.2 Securing places and activities that promote mental health

Theme 2	Sub-theme
Affirming agency	2.1 Maintaining relational support
	2.2 Making meaningful contributions

### Theme One: Finding Meaningful Participation

This theme describes how MHSUs experienced meaningful participation, which supported their recovery journeys. MHSUs and their caregivers described their recovery as an evolving process in which personal change was needed to live with and manage the consequences of their SMHCs.

*I was meeting new friends and learning new skills. Starting to wear new clothes again and travelling on my own to Garden Place* [pseudonym]*- the taste of independence. You must be willing to change.* (Globie)

According to participants, these personal changes involved changing behaviours and social circles that posed an increased risk of relapse. Globie, for instance, reflected on how he needed to cut ties with his friends who used substances.

*It is hard to break away from friends. It is very hard for people to get out of that life. I try. When you are trying to quit, the devil comes to you. So I adapt. Growing up, I went to Sunday school and stuff like that, so that put something in me. That guided me in life, having love for the Lord, you see, there is always something that is pulling you away from trouble and stuff like that.* (Globie)

**Fig. 1:**
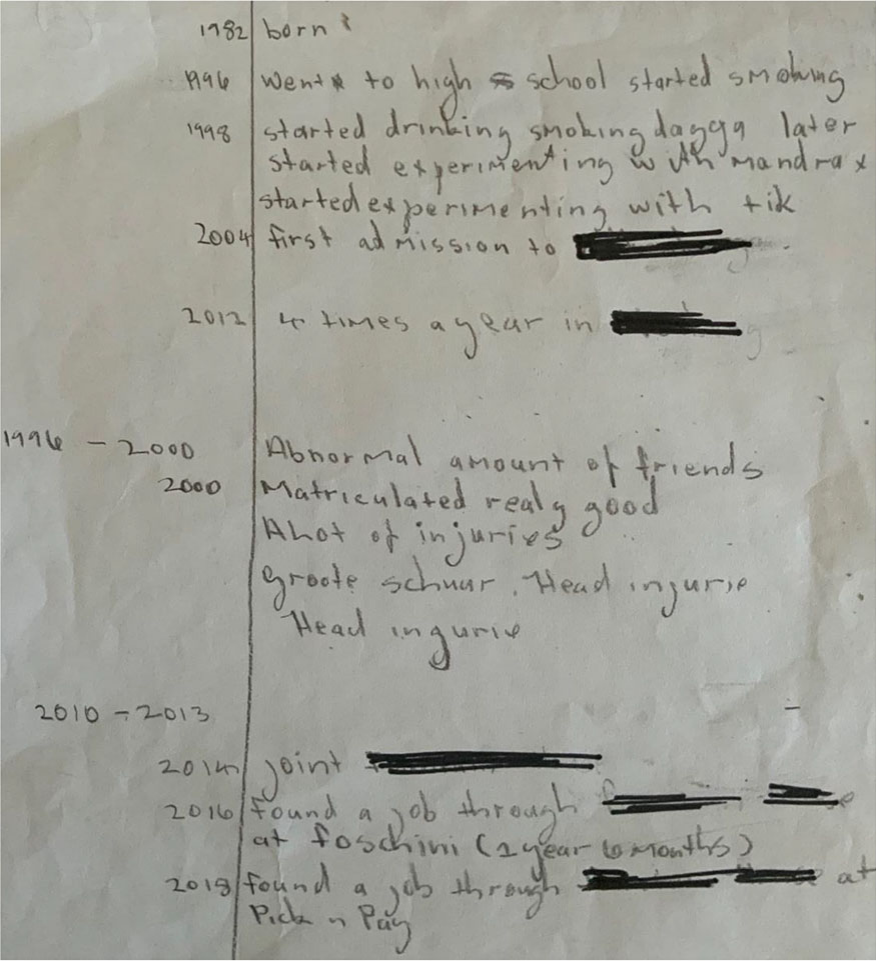
Globie’s life graph showing his significant life events

Globie’s life graph (Fig. 1) offers insights into the significant events related to living with a mental illness. Events referenced on his life graph included: experimenting with drugs when he started secondary school; peer pressure in adolescence; multiple psychiatric hospital admissions; and connecting with an MH-NPO for support. With time, this MH-NPO enabled him to access work opportunities.

MH-NPOs offered psychoeducation to MHSUs and their caregivers as all parties engaged in learning to understand SMHCs and its challenges.

*His dad and I went immediately to a workshop to understand what was wrong with him. He was at home, he had difficulty holding a job, got involved with gangsterism. And the first thing we let him do is start, let him take control of himself, and take his medication. Because at first, I had to give it to him. Every morning, I had to stand in front of him to take it and then eventually we got him to the stage where he went and took the medication himself. He had to take responsibility for what was wrong with him.* (Patrick’s Mom)

Caregivers supported MHSUs as they learnt to cope with their SMHCs. MHSUs and caregivers recognised that knowledge about SMHCs was powerful in demystifying SMHCs, offering insights into how to cope. When caregivers sought knowledge on SMHCs they did so to support and understand MHSUs, and this promoted everyone’s mental health.

**Fig. 2:**
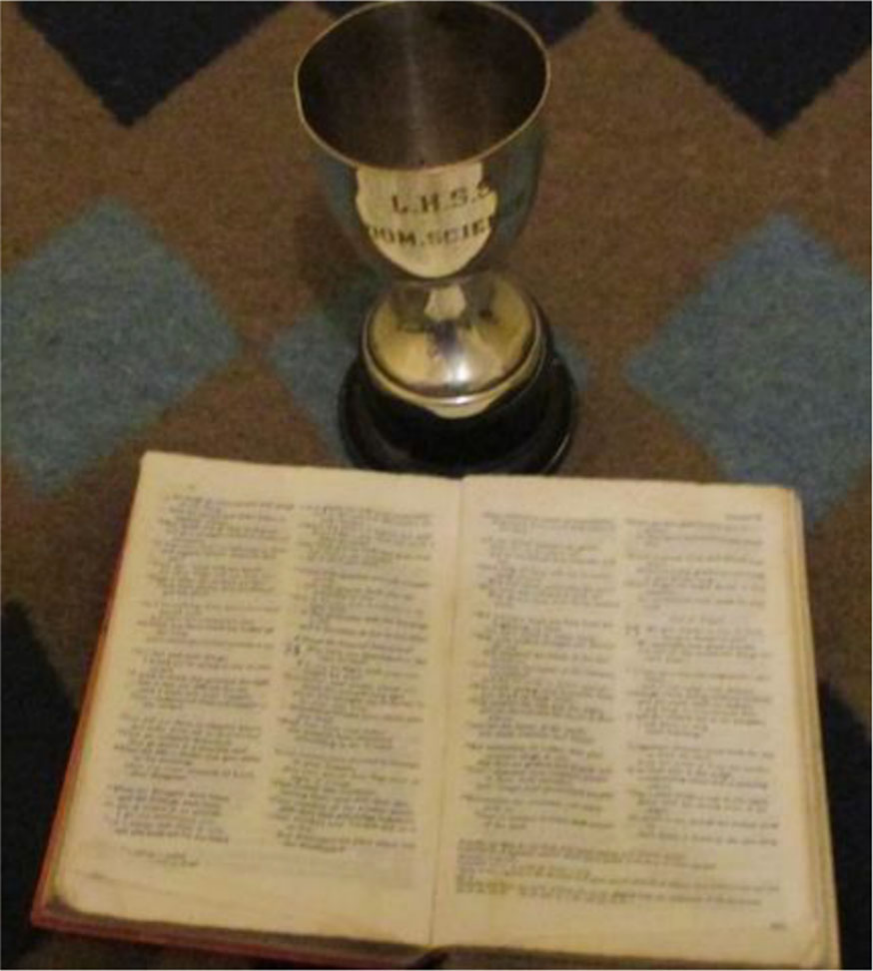
Globie’s photo showing his spiritual practice

Finding meaning included learning to maintain their mental health and accept their SMHCs. MHSUs drew on their spirituality and belief in a Higher Power for sense-making during their recovery journeys (see Fig. 2).

*I think also religion helps. To sustain any endurance that comes before you and not give up when things are too hard for you to overcome.* (Siya)

*God, you must have a plan for me.* (Reggie)

Reflecting on this, Reggie explained that he was first diagnosed with an SMHC at 23 years, and he had multiple clinical relapses and hospital admissions between the ages of 23–29 years old. After three suicide attempts, Reggie decided to trust a Higher Power at the age of 31. He no longer questioned why he had a mental illness; instead, accepting it as part of his life journey.

The diagnosis of SMHC was overwhelming for MHSUs and their caregivers. Despite looking to their religious community for spiritual support, MHSUs and their caregivers reported being ostracised and unsupported by representatives of their religious communities.

Globie’s mother shared her perspective on stigma:

*When I look out of the window, I see religious people laugh at him. My heart was sore. For me, it was a terrible thing. I did not expect it from them. He was broken, dirty, up and down. He was in a bad condition. Religious people laughed at him. Oh, I was so disappointed.* (Globie’s mother)

The stigma surrounding SMHCs was cited as a reason for the lack of support offered to MHSUs and their caregivers by fellow congregants. This led MHSUs to practice their religions on their own.

*I am a person who likes to go to church. But I do not go a lot now, but I love God, the word of God. And I respect the church, but I do not go. I will not lie to you. It is very long since I have gone to church. But what I do is I took a pic of my bible. My bible I read about three times a day.* (Siya)

In the absence of this aid from religious institutions, MHSUs developed self-management strategies (sub-theme 1.1). They identified alternative places and activities to obtain the mental health support they needed (sub-theme 1.2).

### Sub-Theme 1.1: Developing Self-Management Strategies

All MHSUs described developing self-management strategies that they likened to a game plan for staying organised, managing their finances, and diverting their attention from harmful activities that could lead to clinical relapse. Having a game plan also helped them manage their interpersonal relationships.

**Fig. 3:**
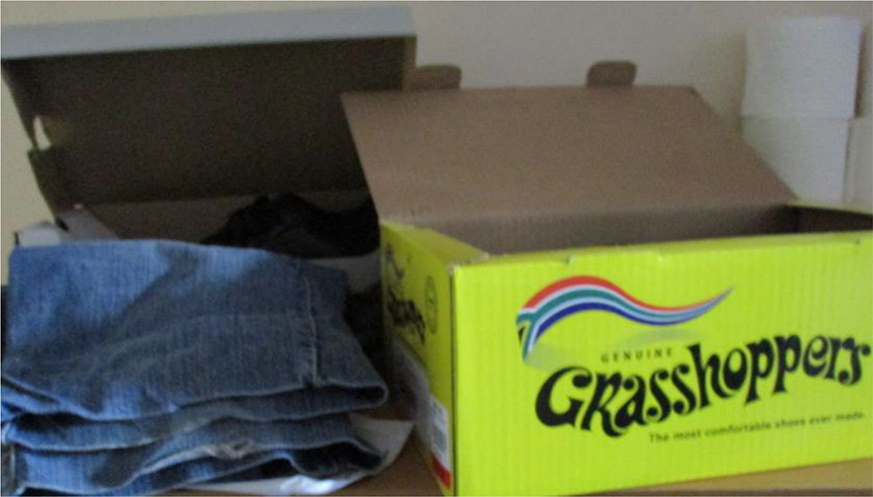
Patrick manages himself by preparing for the workday the night before

Most participants invested in new ways to do their daily activities to maintain good interpersonal relations with others, particularly in communal living spaces. Patrick explained how he carefully organised himself to prepare for his work day (see Fig. 3) to be considerate of others.

While Patrick would not have been bothered with how his morning preparation activities impacted others in the past, it now mattered as maintaining his harmonious living arrangements was important to him. This meant that he carefully selected his clothing every evening to get dressed quietly in the dark every morning.

MHSUs also developed strategies for responding to stigmatising responses to their diagnoses. Some MHSUs dealt with stigma proactively by pre-emptively managing other people’s views. For example, Reggie explained how he makes his diagnosis accessible to others.

*And that is the face of a person with Schizofriendly, not Schizophrenia. A lot of people do not know what Schizophrenia is; they think you are probably crazy. So, they do not get the stigma. You know I tweaked it a little. I would say, I am a Schizofriendly, and they say what is that?* (Reggie)

Reggie and other participants created opportunities to educate others about SMHCs. Participants’ capacities to design and implement their self-management strategy came from incrementally practising self-discipline as opportunities arose. MHSUs also learnt to accept that living with a mental illness requires self-acceptance and self-discipline.

*Having a mental illness is not the end of the world; let your light shine*. *A reward of letting your light shine. The reward is a normal life, and it requires a lot of discipline.* (Globie)

**Fig. 4:**
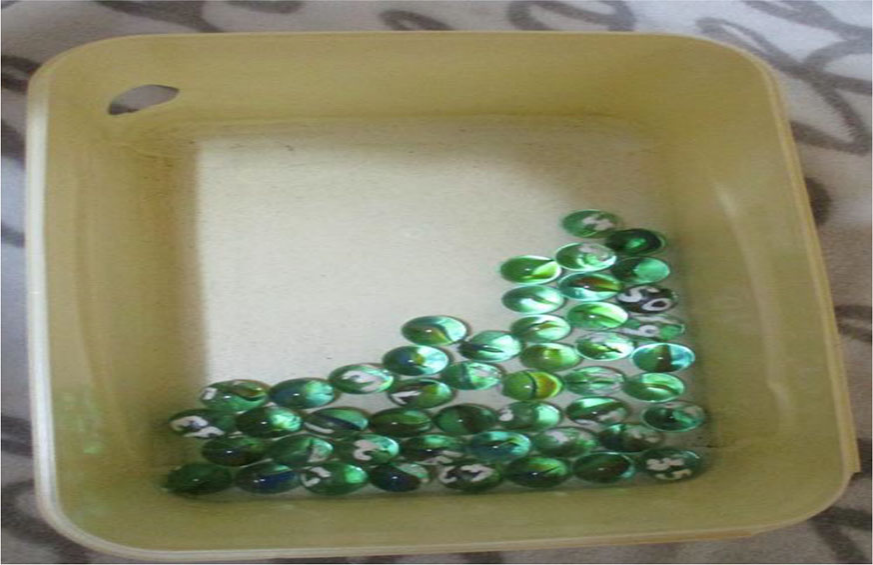
Reggie’s lotto number-generating invention

Another self-management strategy involved financial self-discipline. All participants received a social support grant (1890ZAR/122USD), and some received additional income from casual employment. They budgeted their income carefully to cover basic living costs and some lifestyle choices. For example, Reggie designed a plan to enjoy gambling in a controlled manner. This involved allocating a small amount of money to playing the lottery a few times per month. He created a gambling game where he used marbles with numbers painted onto each marble to randomly select the lottery numbers he would play (see Fig. 4). This gave Reggie a sense of control and helped him resist the temptation to play more often. Reggie explained that:

*I budget myself R160.00 a month, which is R40.00 a week. I do not play horses or anything like that. I do not like how they do the lotto, but I still play because I like playing with the marbles.* (Reggie)

MHSUs also identified alternatives to socialising in environments where drugs and alcohol were readily available. These included watching sports at home with friends or fellow group home residents, enjoying quiet time on their own or creating prayer routines. For example, Globie shared that subscribing to a paid television broadcasting service (DSTV) allowed him to stay home, listen to music, watch movies and socialise with his family.

*The small things like the other day my father would come and watch DSTV with me. Watch a soccer game together. It is the small things that mean a lot to the person with mental illness.* (Globie)

Affording a DSTV subscription also elevated his status in his community. While these self-management strategies were practical, participants revealed that they also built their mental endurance and stamina for new ways of being as part of their recovery journeys.

### Sub-Theme 1.2: Securing Places and Activities That Promote Mental Health

MHSUs drew on different supports to find activities of interest to them. When they felt ready, they found new places and ways of performing their activities. MHSUs described difficulty accessing opportunities to participate in meaningful activities since they were usually only referred to their local clinics to collect their repeat medication prescriptions and only sometimes were informed about MH-NPOs that offered psychosocial support. Consequently, MHSUs and their caregivers only sought assistance from MH-NPOs after many relapses.

**Fig. 5:**
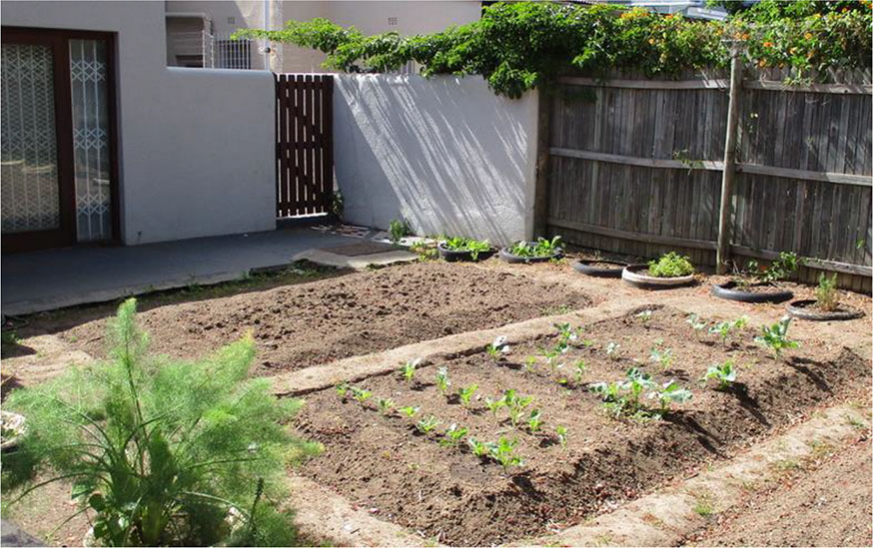
The garden and skills development spaces at Garden Place (Globie)

All the MHSUs in this study described the Garden Place (pseudonym) programme as pivotal to their recovery. MH-NPOs, like Garden Place, offered MHSUs different activities to promote their mental health while also allowing them to develop skills necessary to integrate into society. These services included psychoeducation; supported living in group homes; drop-in services, which MHSUs attend during weekday mornings; skills development programmes; and transitional employment learnerships (see Fig. 5).

MHSUs progressively developed their willingness and confidence to try these activities. Although challenging initially, participating in the MH-NPO programmes opened opportunities and built MHSUs’ confidence.

*First of all, when I started at Garden Place, I was a bit shy. So, it gave me a lot of confidence and positiveness and learning as well. So, I just want to tell you something. I was chairperson, and I went to Johannesburg and Pretoria* [cities outside of the Western Cape Province] *to stand for the rights of people with Schizophrenia and disabilities.* (Siya)

These skills development opportunities allowed MHSUs to try different work opportunities. For example, Reggie held various jobs, eventually securing a job as a project supervisor at a supported employment project run by Garden Place. Garden Place provided a safe place where MHSUs could progress by learning from their experiences, developing new skills, building resilience, being flexible, recognising different possibilities to access networks and utilising available work opportunities. Reggie proudly shared his certificates of completion for courses he accessed through Garden Place.

*A picture on my wall of small achievements. While working for [Health Connected], I was sent on a few courses. I was sent to a first aid course, where I achieved an 80 per cent pass. I also did a business course at UCT Business School. I was sitting there, and the guys were arriving there with their Audis* [luxury vehicle]*. I did it for 6 months. It was a business course. I thought it was okay, but some of the things just went over your head.* (Reggie)

Their caregivers’ encouragement facilitated their uptake of unfamiliar opportunities. Patrick explained how his caregivers' encouraged and motivated him to take advantage of the work opportunities.

*She* (Patrick’s mother) gave me a look as if to say, "you don't wanna f*cking stop. You don't wanna stop. We've had it”. And after a while, they told me, "no, we're not gonna support you anymore". And with that look she gave me, I decided here I better make a change in my life, here I must make a success, here I must survive. I must show these people that I'm worth it, and I can still be a productive member of society, and then they [Garden Place] *got me a job which is very therapeutic, especially when you're a criminal.* (Patrick)

Through their participation at MH-NPOs, MHSUs developed work readiness which assisted them in the relational aspects that MHSUs with SMHCs have difficulty with, as Siya detailed.

*That also empowered me because what I enjoyed most was Garden Place. There's a life skill for everybody, every bloody problem you have on the earth. So, they made me go through many life skills about how to appropriately act in a workplace. When people are rude to you, what are the procedures? Was I work ready? Papers, certificates to prove that you went on a course for work readiness. So, there I learned a lot of life skills and how to cope and defend myself without acting out or acting violently because now you're using knowledge, and knowledge gives you more power, man.* (Siya)

As MHSUs increased their participation in activities offered by MH-NPOs, they required less support from their caregivers and sought opportunities to be more independent. As Patrick's parents described, some MHSUs transitioned from their homes to supported living houses with fellow MHSUs.

Patrick’s mother*: There was always an offer from Garden Place's side that if they wanted to apply, they could apply to go and live in those houses. Patrick never wanted to move away from us. He was going to be here for the rest of his life; mentally, that is what he thought. I kept saying to him; you will have to go live on your own and become independent. You cannot live with us for the rest of your life, but I never forced it. I felt he needed to do it when he was ready.*

Patrick’s father: *He said: “No, I'm not going anywhere! I'm not going to go live on my own. No, I’m not leaving this house!”*

Patrick’s mother*:* [Laughing]* He just wouldn't think of it, and he is so happy now, you know what I mean, that he had taken that step, and as I said, he was about thirty-eight when he took that step to go and, to apply, and he got the place you know. He got a room there.*

Through these opportunities, MHSUs become less reliant on their caregivers for their basic needs and could find new ways of living, working and socialising.

### Theme Two: Affirming Agency

All the MHSUs repeatedly experienced public humiliation associated with their behaviours while they had acute clinical symptoms of SMHCs. Theme two describes how MHSUs overcame this public humiliation and reinvented themselves in their community.

**Fig. 6:**
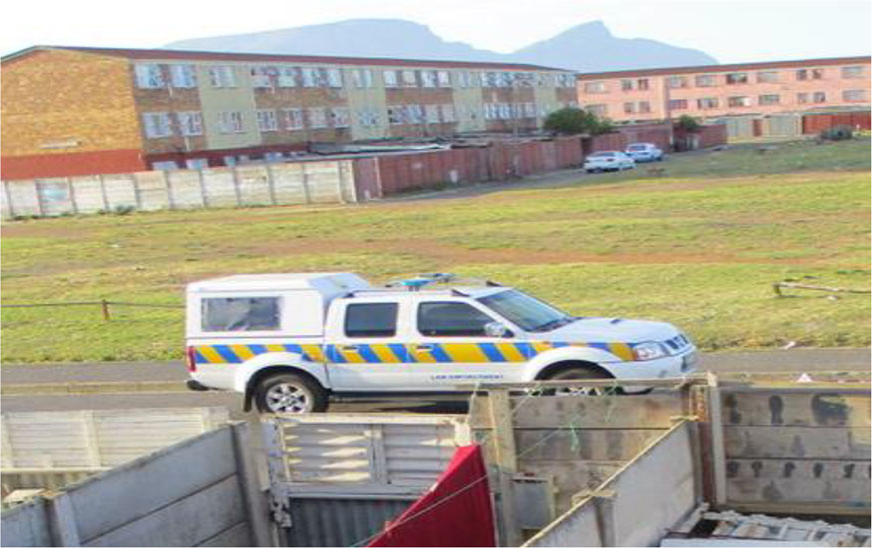
Globie emphasises the role of police, showing a van in front of his gate

All the MHSUs came from Cape Flats communities, with high levels of visible policing due to crime. Most families relied on the police to assist when the MHSUs required hospital admission. This resulted in MHSUs being involuntarily transported to the hospital in a police van. This violent way of containing the MHSU and facilitating their hospital admission often took place in full view of their neighbours. It was traumatic for everyone involved. Globie photographed a police van to show this (Fig. 6).

These interactions with the police when they were acutely ill contributed to MHSUs lingering feeling of not being good enough in the eyes of the public. These humiliating experiences created a need for MHSUs to find new ways of demonstrating and affirming their agency. Being able to show that their life did not stop after a hospital admission was important to MHSUs. Securing a job and owning items such as name-branded clothing and sneakers were a source of pride and a sign of progress for MHSUs.

*Now I have moved from a gangster to a productive member of society. Sometimes I feel proud of the man that I have become. Working has given me financial freedom and has taught me to fend and stand on my own two feet. A job is therapeutic, especially if you are a criminal. A job gives you meaning and purpose.*

(Patrick)

MHSUs developed their sense of agency through relationships with people who promoted their sense of mental well-being (sub-theme 2.1) and by contributing meaningfully to their households and communities (sub-theme 2.2).

### Sub-Theme 2.1 Maintaining Relational Support

MHSUs identified that MH-NPO staff and their caregivers provided them with much needed, ongoing relational support, which helped build resilience despite clinical relapses.

*My mother is always there, and people are there for me. Again, like my social worker, Garden Place, the staff of Garden Place, the managers at work, my supervisors at work. I always have a good relationship with them, which keeps me motivated.* (Globie)

MHSUs and their families did not always have the ongoing support needed to navigate the stigma and trauma associated with the way that involuntary readmissions to a psychiatric hospital occurred. MHSUs recognised that they needed enabling environments that included supportive people for their recovery. Reggie used a photograph of a tree-lined road to explain.

*I think every year was a road for me. Every year, you know, if I had my cycle and got sick, I had to go back, and I believe there is no way back in life. So I am looking forward. I do not really want to go from the pavement into the gutter or something like that*. *Basically, my medication, my support system that I get at home, and my colleagues at work. We are very close, we are like a very close-knit family, but you know, in the work sense.* (Reggie)

People who believed in participants when they had difficulty believing in themselves or gave participants a chance were valued as enablers. For example, Patrick’s psychiatrist's efforts enabled him to access MH-NPO services when they were not offering services to MHSUs in the forensic psychiatry system.

*I would just like to thank Horizon, and I would like to thank Garden Place for giving me a chance and for believing in me because you know Garden Place do not take people with criminal records because it is against their policy, but Dr R wrote such a beautiful letter to them, and they said they would give me a chance.* (Patrick)

**Fig. 7:**
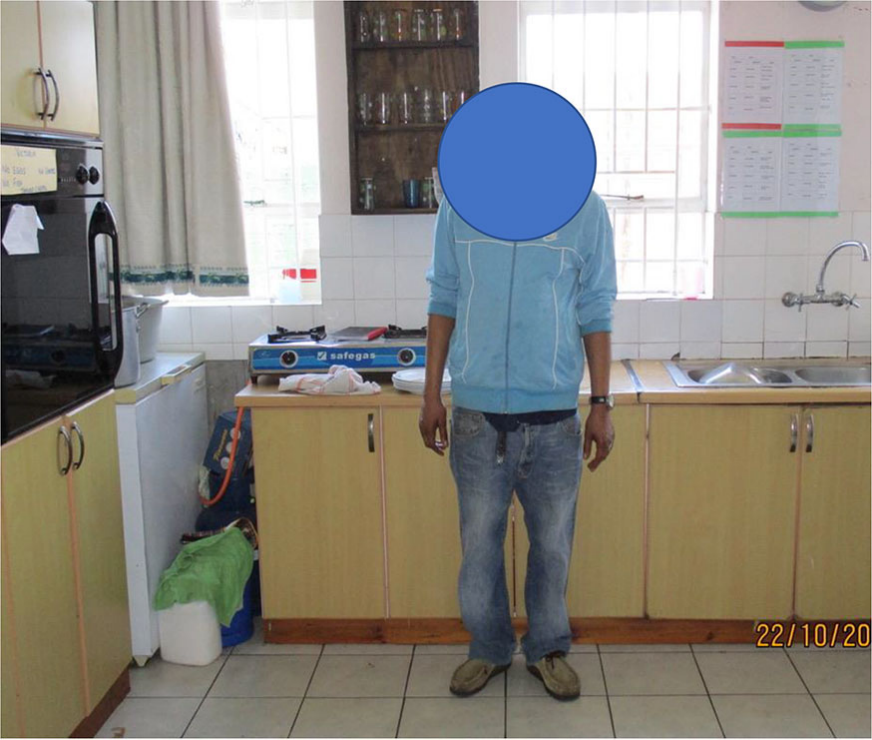
Pictured in the kitchen at the crèche

**Fig. 8:**
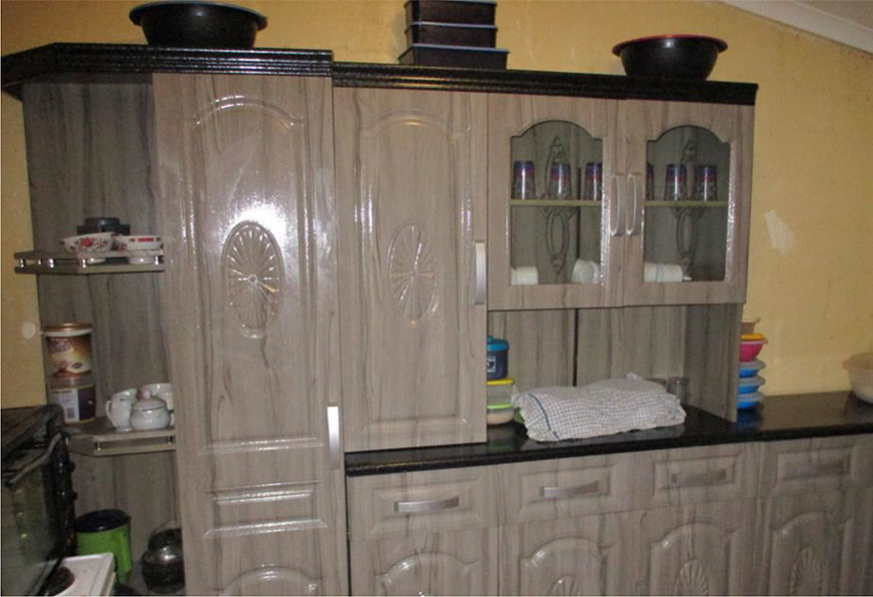
Globie’s picture showcasing the kitchen renovation he paid for in the family home

Having meaningful work and a stable home environment helped participants move forward. Growing and developing in supportive environments allowed MHSUs to have hope in new possibilities and live meaningful lives despite their diagnosis of SMHCs (Figs. 7 and 8).

*With lots of love and the ability to learn, everything is possible (Reggie).*

### Sub-Theme 2.2: Making Meaningful Contributions

By contributing financially or volunteering their services in meaningful ways, MHSUs felt a sense of citizenship. Patrick is a cook at a creche, and his kitchen is his "*pride and joy”.* Patrick's stable living environment, supportive champions, and job contributed to his sense of fulfilment and agency. He felt he added value and positively contributed to the spaces he engaged in.

*The children are beautiful to work with. The one child comes up and says, "thank you, Mom". I said, "I'm not your mom".* [Giggling]. *Because of the food, the Chairperson of the Board says: “and in the kitchen, we have an excellent chef". Children are very honest man. So, you have to know what you're doing, and then you have to put some effort and some love into it, and it's not just about throwing potatoes in a pot. It's done with some sense of pride.* (Patrick)

MHSUs were pleased that, as men, they could assist their parents, siblings and others financially. Globie helped his father and paid for home improvements to their family home and luxury entertainment items. His entire family benefitted from his contributions. By supporting home improvements, MHSUs could showcase their successful recovery to their communities.

When MHSUs could access work, they generously shared what they could with other MHSUs. Patrick explained:

*JP* [a fellow group home resident] *always does me a favour, you know, and I always give him something because he doesn't work. He installed that whole bloody decoder for me. And I gave him 50 rand just to say thank you because he had to climb up the roof to attach it to the dish.* (Patrick)

The theme of Affirming agency captures how MHSUs progress towards living successfully in the public eye. They do this by accessing MH-NPOs support services, which allow them to find their purpose and participate meaningfully as citizens in their chosen communities. Thus, individuals navigated personal recovery in their daily experiences through intrapersonal, interpersonal and group interactions with the rest of their community and society. Through doing well in different spaces, MHSUs were affirmed as people. Their ability to work further supported their recovery. Additionally, MHSUs measured their success by the symbolic status acquired through their material possessions and by contributing to improving the lives of others.

## Discussion

The findings of this study yielded significant insights into how South African men and their caregivers experience personal recovery from SMHCs. First, the stigma faced by MHSUs led them to seek opportunities for support and meaningful participation outside of their community and the social institutions that are traditional sources of support. Second, MHSUs built their capacities for personal recovery by participating in MH-NPO activities which offered them long-term relational support. Third, accessing work opportunities and making meaningful contributions to others is an integral part of the recovery journey for MHSUs. The findings resonated with existing research identifying recovery as an active, complex process grounded in the daily life challenges MHSUs faced (Krupa et al., 2009; Leamy et al., 2011; Mathew et al., 2018; Sommer et al., 2021; Sullivan et al., 2017). The CHIME framework foregrounds the positive and individual aspects of recovery (Stuart et al., 2017), and its optimistic themes do not reflect the difficulties and struggle MHSUs face as part of their recovery process. A focus only on the personal strengths of the individual (Brijnath, 2015) and without due consideration of contextual barriers influencing recovery is reductionist. Ongoing research into the social, economic, cultural and political environments which help or hinder recovery is needed for mental health reform (Gamieldien et al., 2021a, b).

The results of this study make an essential contribution to the CHIME framework from a LMIC perspective. Stigmatising attitudes of others, including from traditional support structures like organised religious structures, limited MHSUs opportunities to engage in meaningful activities in their local community. MHSUs faced stigma due to their community's poor understanding of mental illness. This adversely affected MHSUs and their caregivers, resonating with the dimensions of CHIME, which identifies that a cultural group or a community can either help or hinder recovery (Leamy et al., 2011). In HICs, spirituality is not reported in relation to religion and belonging to a collective through faith-based organisations. Instead, it is framed as an individual practice where MHSUs use their beliefs to make sense of their mental illness (Brijnath, 2015; Jaiswal et al., 2020). This differs from MHSUs in LMICs, BME and ethnic majority groups, where belonging to faith-based communities and having a religious affiliation were essential components of the spiritual aspects of their recovery (Brijnath, 2015; Gamieldien et al., 2021a, b; Leamy et al., 2011). There is a growing interest in using faith-based organisations to offer community-based mental health services (Scheid & Smith, 2021). However in this study, the stigmatising attitudes of congregants in faith-based organisations meant that MHSUs and their caregivers did not view or use these organisations as recovery partners. Instead of relying on their local communities and religious affiliations, the MHSUs in this study sought support from MH-NPOs.

MHSUs and caregivers in this study accessed MH-NPOs, such as those implementing a clubhouse model (Kinn et al., 2018; McKay et al., 2018), because it offered them a sense of community and provided varied, long-term relational support. Similar to other studies, (Bitter et al., 2020; Onken et al., 2007), the support offered by the MH-NPOs facilitated the MHSUs to participate in socialising, learning, living and working environments. The MH-NPOs adopted recovery-oriented practices by addressing the elements of recovery which resonate with the CHIME domains (Leamy et al., 2011). These MH-NPOs provided multi-stakeholder support after discharge from the hospital. A scoping review on recovery from severe mental illness in LMICs (Gamieldien et al., 2021a, b) reported that MHSUs personal recovery mostly drew on similar such community spaces for support. For MHSUs in this study, the MH-NPOs acted as inclusive communities that provided long-term relational support through various services, including access to alternate living arrangements, day programmes and work opportunities. Since their living, learning, socialising and working needs were supported to varying degrees by the MH-NPOs, it is acknowledged that participants could have foregrounded the utility of the MH-NPO in this study. Further studies with MHSUs who are not associated with MH-NPOs will provide insight into possible alternative modes of support.

Supported housing offered by MH-NPOs is an alternative to living with family or alone. When MHSUs socio-economic constraints forced them to live with family, they often experienced an extended childhood (Gamieldien, Galvaan, & Duncan, 2021a). By moving from the family home into communal living, MHSUs in this study experienced success and independence. Moving out of the Cape Flats, and having a place to live separate from family allowed MHSUs to explore new opportunities. Their affiliation with the MH-NPO facilitated their access to resources that developed their skills in the societal, personal, functional, lifestyle and cultural aspects of recovery (Bitter et al., 2020).

Moving out of the Cape Flats, and having a place to live separate from their families allowed MHSUs to explore new opportunities. Independent living is often cited as an essential recovery indicator for HICs, but in LMIC settings it is not as heavily weighted (Gamieldien et al., 2021a, b). For participants in this study, living on their own was not an option due to socio-economic and cultural constraints. Independent living was mostly unaffordable or inaccessible to participants in this study due to socio-economic factors such as low income, and dependence on social grants. Moving out of the family home to live with others was viewed as a step towards independence and gave MHSUs the opportunity to become more autonomous.

Realising a different life meant that MHSUs had to constantly weigh their options and strategies to achieve mental, emotional and spiritual health and well-being (Llewellyn-Beardsley et al., 2019). MHSUs developed insights into how their identity and their views on masculinity influenced their recovery. MHSUs needed to be courageous and follow their path despite setbacks while balancing feeling secure and taking risks (Deegan, 1996). To build their capacities to consistently live purposeful and personally meaningful lives as part of their recovery, MHSUs invested in their personal growth. Adopting a learning spirit and being open to change and new opportunities were notable characteristics of navigating recovery.

As part of their recovery journeys, MHSUs actively resisted peer pressure to engage in health-compromising activities that were prevalent in their poor socio-economic environments. While being patient and tolerant of themselves, they sought alternative places to make connections, such as the MH-NPO, which supported their recovery. The role of context as critical for recovery is well recognised in literature from HICs (Llewellyn-Beardsley et al., 2019; Roe et al., 2020; Leendertse et al., 2021; Slade et al., 2021; Sommer et al., 2021). MHSUs fluctuated in how they exercised choice and autonomy in choosing contexts which either helped or hindered their recovery. A meta-synthesis reporting on the role of place in recovery (Doroud et al., 2018) showed that context was an anchor for daily living in HICS, but contextual factors can have negative or positive outcomes for MHSUs living in LMICs.

The contribution of work to the mental health and well-being of people living with SMHCs is well documented in the literature (Devine et al., 2020). In this study, MHSUs used their ability to work, and contribute to their households to demonstrate masculinity and agency. Opportunities to work in supported employment or the open labour market allowed male MHSUs to find meaning and develop confidence in themselves as contributing members of society. Being tactical when taking up interpersonal and skills development chances from MH-NPOs helped male MHSUs in this study to consider a range of flexible, self-paced learning and employment opportunities. In a LMIC setting like South Africa, with a high unemployment rate, developing entrepreneurial skills would also help MHSUs access more varied occupations (Gamieldien, Galvaan, & Duncan, 2021a; Gamieldien & van Niekerk, 2017).

### Strengths and Limitations of the Study

The research was conducted with persons with lived experience who are part of a hard-to-reach population. Combining visual methods and interviews over multiple data collection sessions allowed numerous opportunities to ensure the research's credibility. The contextual detail offered through the visual techniques would have been difficult to describe if data collection relied only on interviews (Han & Oliffe, 2016). Member checking (Lincoln & Guba, 1985; Nowell et al., 2017; Thomas, 2017) was done by discussing the data presented in the life graph alongside the photographs taken by MHSUs during the final interview. The findings offer some insights on recovery for men with SMHCs and their caregivers and provide possibilities for future research. Limitations of the study were: First, the sample size was small as it was a qualitative study, but rich insights were gained with these experts by experience. Second, the study included MHSUs connected to two MH-NPOs following a clubhouse model based in Cape Town, not to other organisations from other parts of South Africa. As members who had benefited positively from their involvement in the MH-NPO, they might have been more inclined to participate. Third, because of failed recruitment attempts with this hard-to-reach population of people living with SMHCs, all MHSUs were recruited via an MH-NPO. Fourth, due to resource constraints we did not have access to translation services and thus did not set out to recruit MHSUs who were unable to converse in either English or Afrikaans.

## Conclusion

This study has provided insights into the contextual, social, economic, political, demographic, cultural, ethnic, spiritual and intersectional factors which help or hinder recovery from SMHCs. Promoting personal recovery should include a human rights-based approach to mental health service design and delivery which addresses contextual factors, including stigma and social belonging (Desai et al., 2021), while also involving all the stakeholders involved in supporting recovery from SMHCs (Ørjasæter & Almvik, 2022; Sullivan et al., 2017). Understanding of MHSUs experiences is vital to building personal recovery assets and services which address the diverse needs of MHSUs. Scaling up mental health services that include MH-NPOs will enable MHSUs to access services outside the public health sector that supports their recovery in their communities.

## Footnotes

1 Coloured identity refers to people with mixed heritage and creolised identities shaped and re-shaped by historical practices in South Africa Erasmus, Z. (2000). Recognition through pleasure, recognition through violence: Gendered coloured subjectivities in South Africa. *Current sociology*, *48*(3), 71–85. 10.1177/0011392100048003006.

2 Due to resource constraints a translator was not involved in the study. However, this did not preclude MHSUs who spoke isiXhosa from participating as all MHSUs who were approached could communicate in English or Afrikaans.

## Declarations

### Paper Context

To our knowledge, there are limited qualitative studies on service user views on personal recovery from SMHCs in South Africa. However, the findings suggest that mental health service transformation must involve service user and caregiver perspectives to inform recovery-based services.
